# Feasibility of Smartphone-Based Markerless Motion Capture for Quantitative Gait Assessment in Pediatric Guillain–Barré Syndrome: A Two-Case Proof-of-Concept Study

**DOI:** 10.3390/bioengineering13010027

**Published:** 2025-12-26

**Authors:** Yu-Sun Min

**Affiliations:** 1 Department of Rehabilitation Medicine, School of Medicine, Kyungpook National University, Daegu 41944, Republic of Korea; ssuni119@knu.ac.kr; 2 Department of Rehabilitation Medicine, Kyungpook National University Chilgok Hospital, Daegu 41404, Republic of Korea; 3 AI-Driven Convergence Software Education Research Program, Graduate School of Computer Science and Engineering, Kyungpook National University, Daegu 41566, Republic of Korea

**Keywords:** gait analysis, Guillain-Barré syndrome, markerless motion capture, neuromuscular recovery, OpenCap, pediatric rehabilitation

## Abstract

This two-case proof-of-concept study evaluated the feasibility and clinical utility of a smartphone-based markerless motion capture system for quantitative gait assessment in pediatric Guillain–Barré syndrome (GBS). Two children with GBS underwent overground gait analysis using a dual-smartphone setup (OpenCap), and three-dimensional hip, knee, and ankle kinematics were computed via OpenSim. Case 1, a boy with treatment-related fluctuation, demonstrated marked abnormalities in swing-phase limb advancement and ankle push-off that improved after six weeks of rehabilitation in parallel with gains in muscle strength, balance, and ambulation. Case 2, a girl recovering from acute inflammatory demyelinating polyneuropathy, exhibited residual reductions in hip and knee flexion and impaired ankle control despite normal strength, consistent with vestibular dysfunction. All assessments were completed within routine clinical time constraints and produced analyzable kinematic data using only two smartphones. These findings indicate that smartphone-based markerless motion capture is a feasible and informative method for detecting gait impairment and recovery patterns in pediatric GBS and may serve as an accessible digital biomarker to complement standard clinical evaluations.

## 1. Introduction

Guillain–Barré syndrome (GBS) is an acute immune-mediated polyneuropathy that commonly results in weakness, balance impairment, and gait disturbance in children [1,2]. Although most pediatric patients eventually recover functional mobility, the trajectory of neuromuscular recovery varies widely across individuals and is influenced by the degree of motor involvement, sensory dysfunction, and rehabilitation intensity [3,4,5,6,7]. In clinical practice, recovery is typically tracked using subjective or low-resolution tools such as the Medical Research Council (MRC) score, Functional Ambulation Category (FAC), Berg Balance Scale (BBS), and simple timed walking tests [8,9]. While these measures are practical, they offer limited insight into the biomechanics of gait or the underlying mechanisms of residual impairment, particularly when muscle strength has partially or fully normalized [10].

Objective gait analysis can provide valuable information on joint kinematics, inter-limb coordination, and compensatory strategies that are not detectable through standard clinical scales [10,11,12]. However, access to quantitative gait biomechanics is often limited by the cost, complexity, and space requirements of laboratory-based motion capture systems, which are rarely available in pediatric rehabilitation settings [6,10]. This gap is especially problematic in pediatric GBS, where subtle abnormalities—such as impaired ankle push-off, asymmetric swing mechanics, or vestibular-driven instability—may persist despite normal muscle strength and can influence long-term mobility outcomes. Therefore, accessible tools that offer quantitative yet clinically feasible gait analysis are needed.

Advances in computer vision have enabled markerless motion capture systems capable of extracting three-dimensional human movement data from smartphone videos [6,13,14,15]. Several markerless motion capture platforms have been developed in recent years, including commercial systems such as Theia3D and research-oriented frameworks such as DeepLabCut [16]; however, many of these approaches remain limited by cost, technical complexity, or restricted suitability for routine clinical use, motivating the growing interest in accessible smartphone-based solutions such as OpenCap. OpenCap, a recently developed, open-access platform, integrates multi-camera video capture with cloud-based pose estimation and musculoskeletal modeling, enabling rapid quantification of kinematics without markers or specialized hardware [17]. Its low cost and minimal setup requirements make it particularly appealing for pediatric rehabilitation environments, where engagement, time efficiency, and minimal intrusion are critical [18]. While OpenCap has been validated in healthy adults and certain sports and clinical tasks, its feasibility and clinical utility in pediatric neurological populations remain largely unexplored.

This two-case proof-of-concept study aimed to evaluate the feasibility and clinical utility of smartphone-based markerless motion capture for quantitative gait assessment in pediatric patients with GBS. By analyzing two children with distinct clinical profiles—one with treatment-related fluctuation undergoing longitudinal rehabilitation and one with subacute AIDP presenting with vestibular-related imbalance—we sought to demonstrate how markerless kinematics can supplement conventional clinical assessments, characterize residual deficits, and capture recovery-related changes. These preliminary findings may inform future work toward integrating digital gait biomarkers into pediatric neurorehabilitation. The primary objective of this study was to evaluate both the feasibility and clinical utility of a smartphone-based markerless motion capture system for quantitative gait assessment in pediatric Guillain–Barré syndrome (GBS). Feasibility was defined a priori as the ability to deploy the system within routine pediatric rehabilitation settings, without specialized laboratory infrastructure, while achieving reliable gait reconstruction in children with neurological impairment. Clinical utility was defined as the system’s capacity to identify clinically interpretable, joint- and phase-specific gait deviations relative to age-matched normative data and to generate outputs that could inform individualized rehabilitation planning. By explicitly defining feasibility metrics and clinical endpoints, this study aimed to move beyond descriptive demonstration and provide a structured proof-of-concept evaluation of smartphone-based markerless gait analysis in pediatric neurorehabilitation.

## 2. Materials and Methods

### 2.1. Study Design and Participants

This study was designed as a two-case proof-of-concept feasibility investigation evaluating the clinical applicability of smartphone-based markerless motion capture in pediatric Guillain–Barré syndrome (GBS). Two pediatric patients with GBS were recruited consecutively from the inpatient and outpatient pediatric rehabilitation program of a tertiary referral hospital between 2023 and 2025. During this recruitment window, seven pediatric patients with a confirmed diagnosis of GBS were screened for eligibility. Inclusion criteria were: (1) age < 18 years, (2) confirmed diagnosis of GBS based on clinical and electrophysiological criteria, (3) ability to ambulate independently or with minimal assistance for at least 5 m, and (4) sufficient cooperation to complete gait recording. Exclusion criteria included severe cognitive impairment, inability to ambulate, use of rigid orthoses or walking aids during assessment, and concurrent orthopedic or neurological conditions affecting gait.

Of the seven screened patients, four met the inclusion criteria, and two completed full markerless gait acquisition without technical failure, forming the basis of this proof-of-concept analysis. Case 1 was a 12-year-old boy experiencing treatment-related fluctuation (TRF) after initial intravenous immunoglobulin (IVIG) therapy. Case 2 was an 11-year-old girl with acute inflammatory demyelinating polyneuropathy (AIDP) who presented with persistent imbalance despite near-normal limb strength. Both assessments were conducted during the subacute recovery phase, at approximately 6–8 weeks after symptom onset, and both participants were able to ambulate independently or with minimal assistance without assistive devices during data collection. Written informed consent was obtained from the parents or legal guardians, and the study was approved by the institutional review board (protocol code KNUCH 2025-09-063).

### 2.2. Markerless Motion Capture System (OpenCap)

Gait assessments were conducted using OpenCap platform (https://www.opencap.ai), an open-access, smartphone-based markerless motion capture platform. Video data were acquired using two Apple iPads (iPad Air, Apple Inc., Cupertino, CA, USA), each equipped with a rear-facing camera recording at a frame rate of 60 Hz. The devices were positioned approximately 4–5 m apart at a height of ~1.0 m and oriented to capture synchronized sagittal and frontal views of overground walking, with camera yaw angles of approximately 30° relative to the walking direction. Recordings were performed in a standard indoor clinical corridor under uniform ambient lighting conditions, without additional lighting equipment. Participants walked at a self-selected speed along a 4–5 m walkway while wearing their usual footwear. System calibration was performed using a standardized five-second T-pose prior to each recording session. Video data were uploaded to the OpenCap cloud environment, where three-dimensional joint center trajectories were estimated using a deep learning–based pose estimation model with multi-view triangulation. No manual landmark correction was performed. Trials were visually inspected, and recordings with substantial markerless tracking failure or severe occlusion were excluded from further analysis. Processing time per trial was approximately 3–5 min.

### 2.3. Kinematic Processing and Inverse Kinematics in OpenSim

Markerless three-dimensional joint center trajectories exported from OpenCap were imported into OpenSim version 4.5 (Stanford University, Stanford, CA, USA) for musculoskeletal modeling and inverse kinematics (IK) analysis [19]. Inverse kinematics was performed using a scaled musculoskeletal model (Lai-Uhlrich, 2022), which is provided as the default OpenSim model within the OpenCap pipeline [20,21,22]. Model scaling was automatically performed using participant-specific anthropometric parameters estimated by OpenCap, including body height and segment length information.

Inverse kinematics was solved using a weighted least-squares optimization to minimize discrepancies between the experimental markerless joint centers and corresponding virtual model markers, according to the following objective function:

$$\underset{q\left(t\right)}{\min}\sum_{i=1}^{N}w_{i}{\left(x_{i}^{exp}\left(t\right)-x_{i}^{model}\left(q\left(t\right)\right)\right)}^{2}$$

where $q\left(t\right)$ represents the vector of joint angles at time t, $x_{i}^{exp}\left(t\right)$ denotes the experimentally estimated markerless joint center positions, $x_{i}^{model}\left(q\left(t\right)\right)$ denotes the corresponding model-predicted positions, and $w_{i}$ represents the weighting assigned to each joint. Uniform weighting was applied across joints, and kinematic analysis focused on sagittal-plane motion of the hip, knee, and ankle to reduce sensitivity to potential frontal- and transverse-plane tracking errors. Joint angle trajectories were low-pass filtered using a fourth-order Butterworth filter with a cutoff frequency of 6 Hz prior to further analysis.

**Figure 1:**
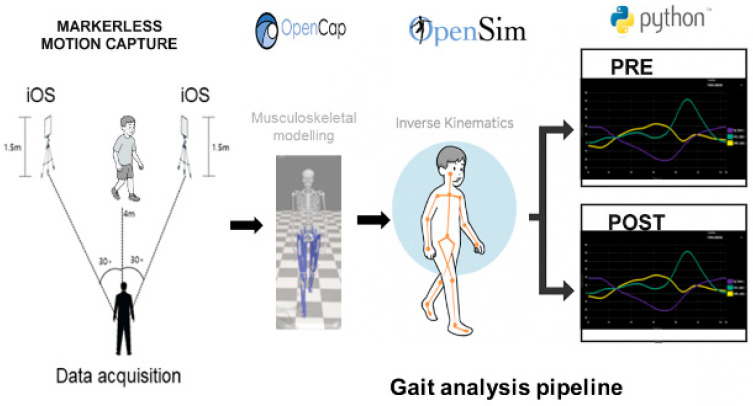
Conceptual framework of the study. The schematic illustrates the workflow for quantitative monitoring of gait recovery in pediatric Guillain–Barré syndrome (GBS) using a smartphone-based markerless motion capture system (OpenCap). Two smartphones positioned at 30° angles capture synchronized walking trials, which are processed through the OpenCap cloud pipeline for three-dimensional pose estimation and inverse kinematics. Resulting joint angle trajectories are compared before and after rehabilitation, enabling visualization of progressive normalization in gait kinematics and symmetry throughout recovery.

For each participant, one representative gait cycle was extracted and time-normalized to 0–100%. Peak joint flexion/extension, range of motion (ROM), and temporal characteristics (peak timing) were calculated. To contextualize deviations, each waveform was plotted against age-matched normative pediatric gait data, with ±1 SD shading (Figure 1).

An analyzable gait cycle was operationally defined as a complete stride in which three-dimensional joint center trajectories were continuously reconstructed throughout the gait cycle without major tracking loss or severe occlusion. OpenCap does not provide a single numerical confidence score for gait cycles; therefore, trial quality was assessed through visual inspection of reconstructed joint trajectories. Trials exhibiting discontinuities, anatomically implausible joint motion, or substantial loss of keypoints were excluded from analysis. For each participant, at least one gait cycle meeting these criteria was identified and included.

The present analysis focused on sagittal-plane kinematics of the hip, knee, and ankle, as these parameters are most directly related to clinically relevant aspects of gait recovery in pediatric GBS, including limb advancement, joint range of motion, and push-off mechanics. Although coronal and transverse plane kinematics were available and visually inspected, these planes showed greater variability and lower signal-to-noise ratio in the current clinical recording environment and did not provide additional clinically interpretable information in these cases. Accordingly, sagittal-plane analysis was selected to enhance robustness and clinical interpretability in this proof-of-concept study.

### 2.4. Clinical Assessments

Clinical measures were obtained on the same day as gait recordings. Motor strength was evaluated using the Medical Research Council (MRC) Sum Score. Functional independence was assessed using the Korean Modified Barthel Index (K-MBI). Balance and mobility were evaluated using the Berg Balance Scale (BBS), Functional Ambulation Category (FAC), and timed performance measures including the 10-Meter Walk Test (10MWT) and Timed Up and Go (TUG), both performed at a self-selected walking speed. For Case 2, vestibular integrity was additionally examined with video-oculography (VOG), bithermal caloric testing, and vestibular-evoked myogenic potentials (VEMP) to explain persistent imbalance.

Case 1 underwent clinical and kinematic assessment at two timepoints (pre- and post-rehabilitation), while Case 2 was assessed at one timepoint during subacute recovery.

### 2.5. Feasibility Evaluation

Feasibility was considered successful when the smartphone-based markerless motion capture system could be deployed within routine pediatric rehabilitation settings while meeting predefined operational criteria. Specifically, feasibility required (1) a setup time of 10 min or less, including camera placement and system calibration, and (2) successful reconstruction of at least one complete gait cycle per participant. In addition, feasibility required (3) completion of data acquisition without adverse events, such as falls, near-falls, or premature termination due to fatigue, (4) operation without the need for wearable sensors, physical markers, or laboratory-grade equipment, and (5) data acquisition performed in a standard clinical corridor without environmental modification. These predefined thresholds were selected to reflect realistic workflow constraints commonly encountered in pediatric rehabilitation clinics and inpatient wards.

### 2.6. Clinical Utility Endpoints

Clinical utility was evaluated using predefined kinematic endpoints derived from joint-angle trajectories of the hip, knee, and ankle across the gait cycle. Clinical utility was considered meaningful if the markerless motion capture system could (1) detect joint-angle deviations exceeding an absolute Z-score of 1.96 relative to age-matched normative trajectories, corresponding to the 95% confidence interval, (2) localize these deviations to specific gait phases, such as stance or swing, (3) reveal patient-specific gait patterns that were not apparent from group-level statistics alone, and (4) provide clinically interpretable outputs that could be mapped to modifiable rehabilitation parameters, including swing-phase assistance, push-off support, or joint-specific strengthening focus. Z-scores were computed pointwise across the normalized gait cycle using the normative mean and standard deviation at each time point, allowing individualized deviation profiles to be interpreted within a standardized reference context.

### 2.7. Normative Dataset and Z-Score Computation

Age-matched pediatric normative gait data were not available at the time of this study because OpenCap is a recently released platform and does not yet provide age-stratified pediatric datasets. Therefore, normative reference trajectories were derived from a dataset of 30 healthy adults collected using the same OpenCap system, camera configuration, walkway length (4–5 m), and self-selected walking speed protocol as those used for patient assessments. From a developmental gait perspective, children aged 11–12 years typically exhibit mature gait patterns that closely approximate adult sagittal-plane joint kinematics and temporal gait structure. Accordingly, adult normative data were used as a pragmatic reference to contextualize joint-angle deviations and recovery-related changes in this proof-of-concept study. For the normative dataset, 3–5 consecutive gait cycles were recorded per participant and time-normalized to 101 points over the gait cycle. Gait cycles were first averaged at the subject level, and group-level normative mean and standard deviation waveforms were computed at each time point. Pointwise Z-scores were calculated using the following equation:

$$Z\left(t\right)=\frac{x_{\mathrm{patient}}\left(t\right)-\mu_{\mathrm{norm}}\left(t\right)}{\sigma_{\mathrm{norm}}\left(t\right)}$$

where μ_norm(t) and σ_norm(t) represent the adult normative mean and standard deviation, respectively. An absolute Z-score threshold of 1.96 was used to indicate deviations exceeding the 95% normative confidence interval.

### 2.8. Data Analysis

Descriptive statistics were used given the small sample size and proof-of-concept design. Kinematic waveforms, peak joint metrics, and step-length symmetry were visualized using Python 3.9. Comparisons between cases and normative data were performed qualitatively. No inferential statistics were applied, consistent with feasibility study methodology.

## 3. Results

### 3.1. Case 1: Longitudinal Gait Recovery Following Rehabilitation

Case 1 was a 12-year-old boy with treatment-related fluctuation (TRF) following initial intravenous immunoglobulin (IVIG) therapy. Prior to disease onset, he was fully independent in daily activities and school participation, with no pre-existing neurological or musculoskeletal conditions. Relevant comorbidities included obesity and non-alcoholic fatty liver disease, which were managed concurrently during rehabilitation. At baseline, the patient presented with proximal lower-limb weakness (MRC 3–4/5), impaired balance (BBS 49/56), and reduced functional independence (K-MBI 60). He was able to ambulate independently without assistive devices at the time of gait assessment, although endurance was reduced and balance was impaired. His gait pattern was characterized by decreased hip and knee flexion during swing, reduced ankle dorsiflexion during stance progression, and asymmetric step lengths. The 10-Meter Walk Test (10MWT) required 18.4 s, consistent with limited community ambulation. Following six weeks of inpatient rehabilitation and a second IVIG cycle, clinical outcomes improved substantially. Lower-limb strength normalized to MRC 5/5, K-MBI increased to 97, BBS reached 56/56, and FAC improved from 4 to 5. Walking speed improved (10MWT: 12.1 s), and TUG performance normalized.

**Figure 2:**
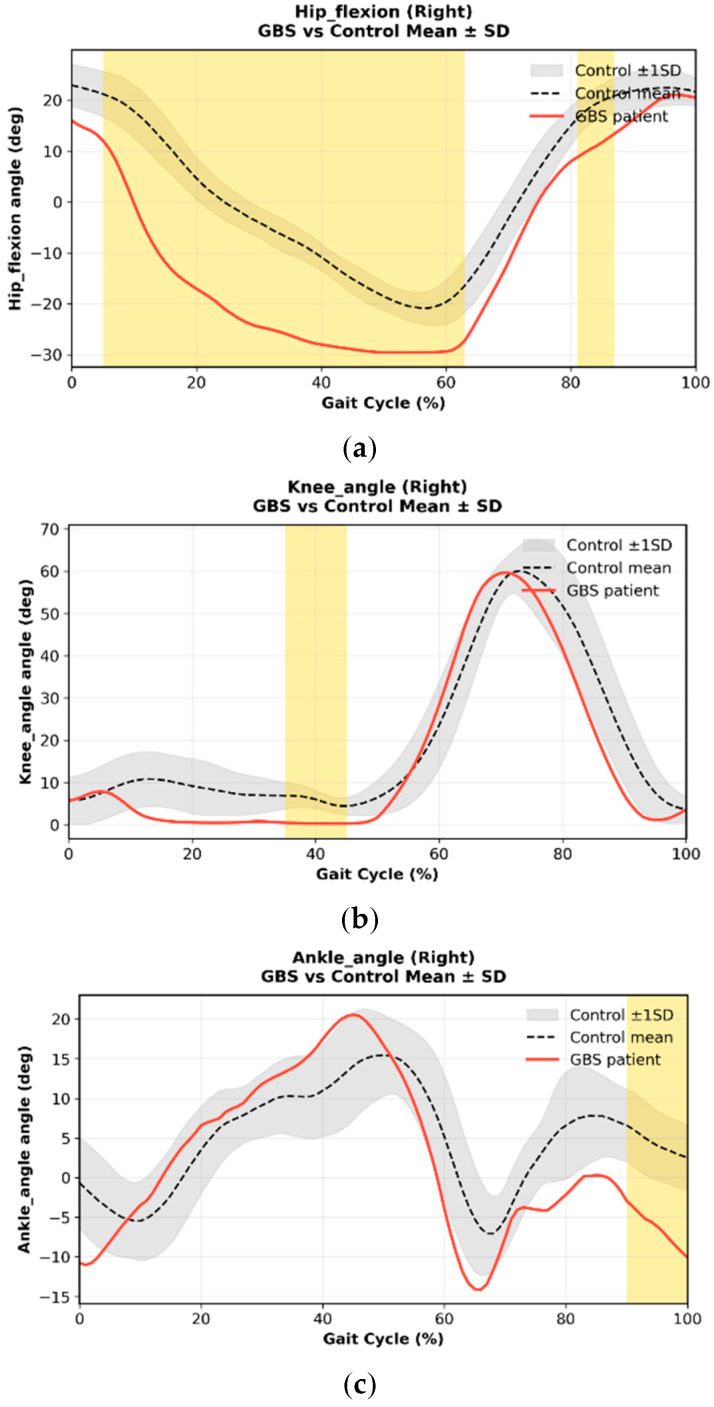
Sagittal-plane right lower-limb joint kinematics in Case 1 at baseline (pre-rehabilitation) compared with normative reference data. Panels show (**a**) hip flexion, (**b**) knee angle, and (**c**) ankle angle across the normalized gait cycle (0–100%). The black dashed line represents the normative mean trajectory, and the gray band indicates the ±1 standard deviation (SD) range. The red line denotes the patient’s kinematic profile. Yellow-shaded regions indicate time points at which the patient’s trajectory deviated beyond the 95% normative confidence interval (|Z| ≥ 1.96), based on pointwise Z-score computation as described in the Methods. Relative to the normative reference, the patient exhibited reduced hip and knee flexion during swing, as well as diminished ankle dorsiflexion and push-off during late stance and pre-swing, consistent with impaired limb advancement and reduced distal propulsion in early GBS recovery.

**Figure 3:**
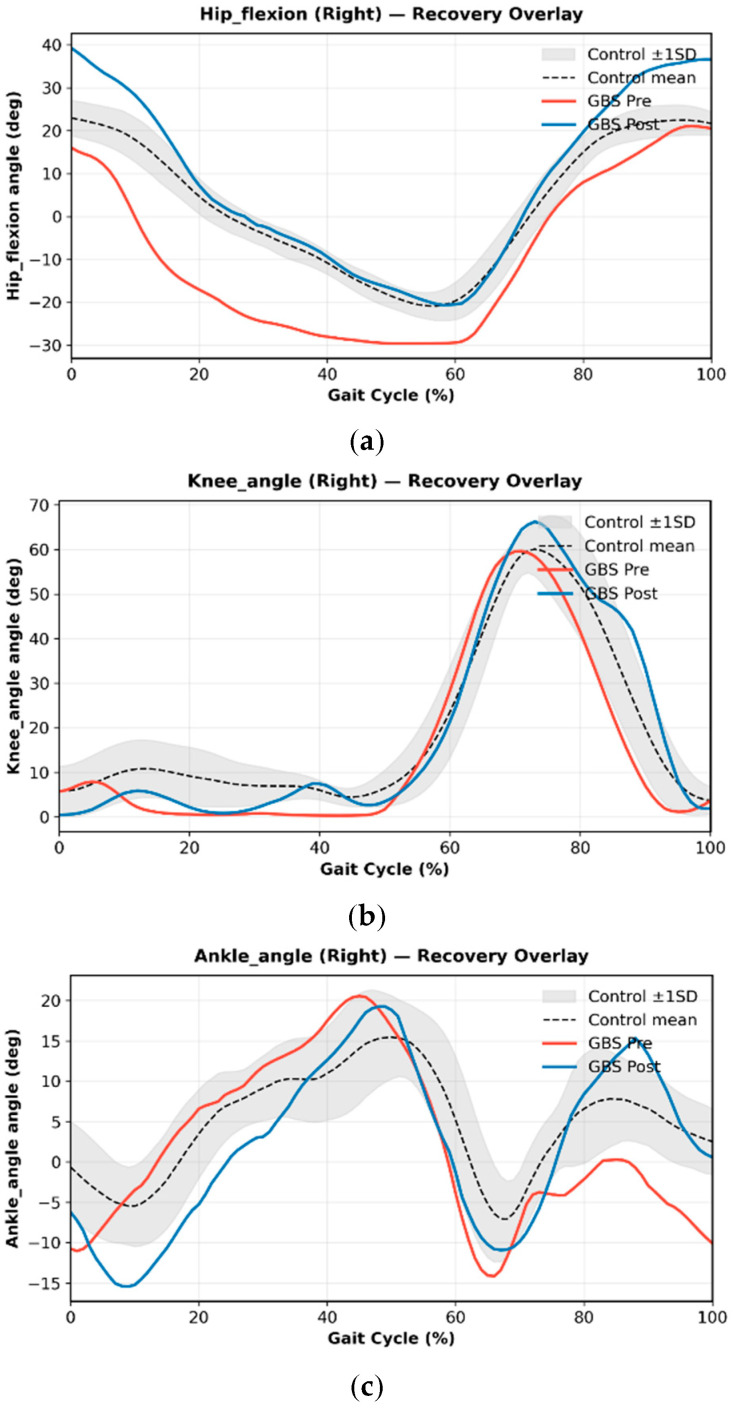
Recovery overlay of right lower-limb joint kinematics in a pediatric Guillain–Barré syndrome (GBS) patient before and after rehabilitation. Panels show (**a**) hip flexion, (**b**) knee angle, and (**c**) ankle angle across the normalized gait cycle (0–100%). The black dashed line and gray shaded band represent the mean and ±1 SD range of healthy controls. Red and blue lines denote the patient’s pre-rehabilitation (GBS Pre) and post-rehabilitation (GBS Post) trajectories, respectively. Following six weeks of rehabilitation and a second IVIG cycle, the patient demonstrated greater hip and knee flexion during swing and improved ankle rocker function, with post-rehabilitation curves shifting toward the normative envelope. These kinematic changes indicate enhanced limb advancement, restored push-off mechanics, and more coordinated swing-phase control, aligning with concurrent improvements in strength, balance, and functional ambulation measures.

**Figure 4:**
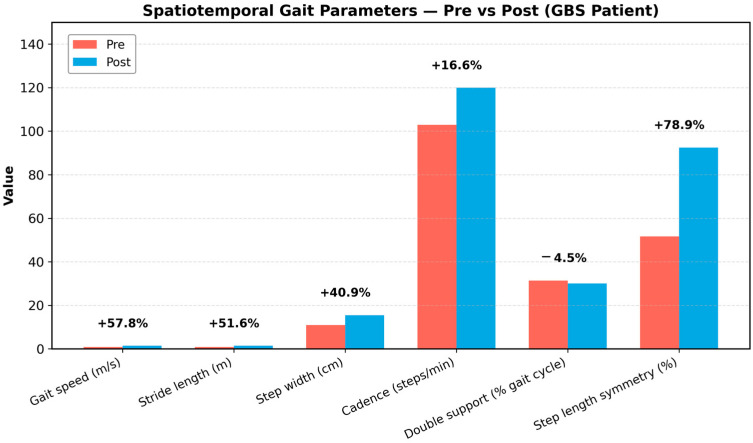
Comparison of spatiotemporal gait parameters before and after rehabilitation in a pediatric patient with Guillain–Barré syndrome (GBS). Bars represent pre-rehabilitation (red) and post-rehabilitation (blue) values for gait speed, stride length, step width, cadence, double-support duration, and step-length symmetry. Percentage changes above each pair of bars indicate relative improvement between sessions. Following rehabilitation, gait speed (+57.8%), stride length (+51.6%), step width (+40.9%), cadence (+16.6%), and step-length symmetry (+78.9%) increased, while double-support duration decreased (−4.5%), demonstrating enhanced gait efficiency and symmetry after neuromuscular recovery.

Case 1 showed clear abnormalities across hip, knee, and ankle motion relative to normative pediatric gait. As shown in Figure 2, hip flexion during early–mid swing (approximately 0–45% of the gait cycle) and terminal swing (70–90%) was markedly reduced, limiting limb advancement. Knee flexion during swing was similarly blunted, with a delayed peak around 60–75%. Ankle motion demonstrated insufficient plantarflexion during terminal stance and pre-swing (50–65%) and reduced dorsiflexion in late stance (60–80%), indicating weak push-off and diminished transition into swing. These patterns reflect proximal weakness and impaired dynamic control consistent with his early clinical presentation. After six weeks of rehabilitation and a second IVIG cycle, the patient showed substantial kinematic recovery (Figure 3). Hip and knee flexion during swing increased toward normative peaks, and ankle rocker function improved across terminal stance and pre-swing. Correspondingly, spatiotemporal parameters demonstrated significant gains in gait speed, stride length, cadence, and step-length symmetry (Figure 4), indicating more efficient, symmetric, and confident ambulation. Taken together, Case 1 demonstrated a mechanically inefficient gait pattern dominated by weak limb advancement and reduced push-off, which improved in parallel with clinical gains in strength, balance, and functional mobility. These findings support the utility of markerless motion capture for tracking rehabilitation-induced recovery in pediatric GBS.

### 3.2. Case 2: Cross-Sectional Characterization During Subacute Recovery

Case 2 was an 11-year-old girl with acute inflammatory demyelinating polyneuropathy (AIDP) who presented with persistent imbalance during the subacute recovery phase. Prior to illness, she was functionally independent with normal developmental milestones and no relevant medical comorbidities. At the time of assessment, she was able to ambulate independently without assistive devices but continued to report dizziness and unsteadiness during dynamic activities despite near-normal lower-limb strength. On clinical examination, lower-limb strength was near normal (MRC 5/5), while balance-related symptoms persisted. Clinical mobility tests revealed mild residual slowing, with a 10-Meter Walk Test (10MWT) time of 12.8 s and a Timed Up and Go (TUG) time of 10.3 s, whereas the Berg Balance Scale (BBS) score had normalized. Vestibular evaluation demonstrated smooth pursuit deficits, left canal paresis, and otolith dysfunction, providing a physiological basis for the observed gait instability.

**Figure 5:**
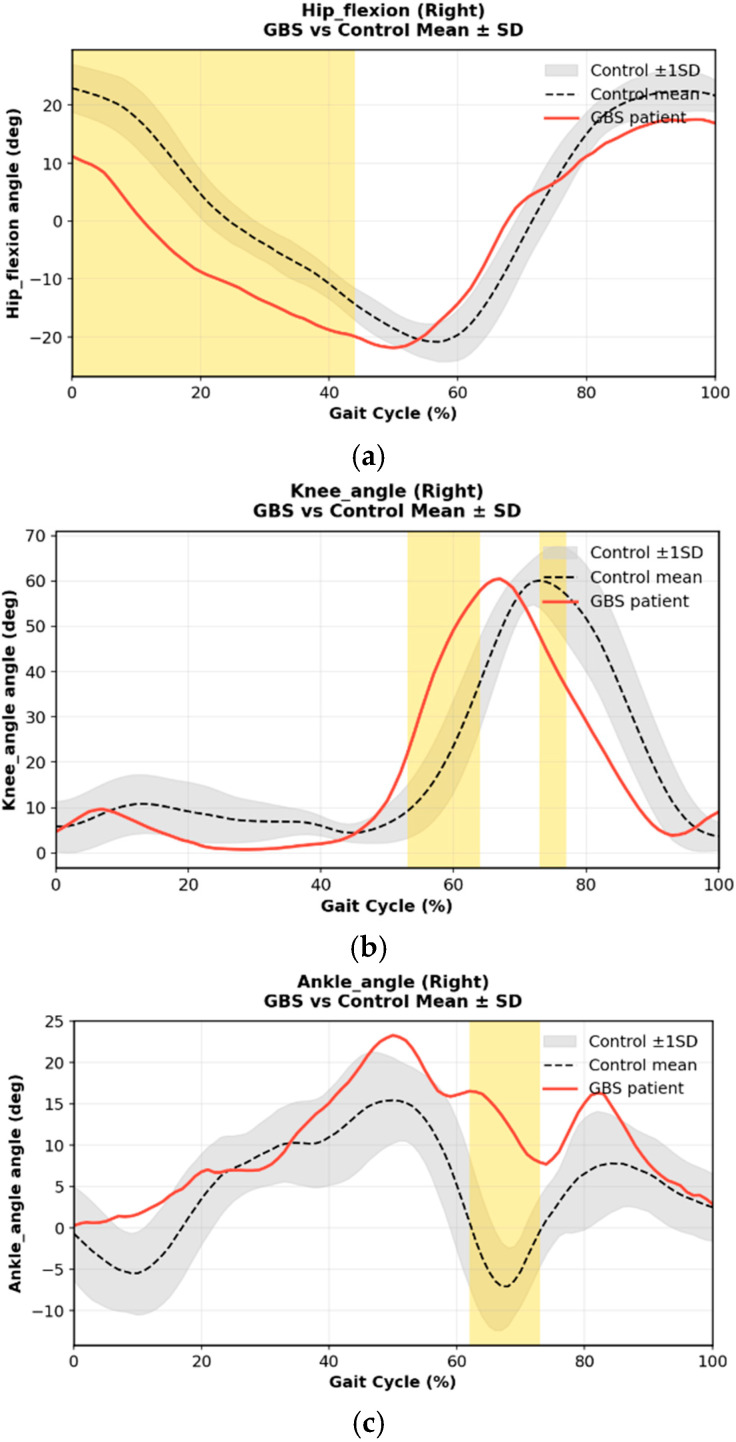
Sagittal-plane right lower-limb joint kinematics in Case 2 during the subacute recovery stage compared with normative reference data. Panels show (**a**) hip flexion, (**b**) knee angle, and (**c**) ankle angle across the normalized gait cycle (0–100%). The black dashed line represents the normative mean trajectory, and the gray band indicates the ±1 standard deviation (SD) range. The red line denotes the patient’s kinematic profile. Yellow-shaded regions indicate time points at which the patient’s trajectory deviated beyond the 95% normative confidence interval (|Z| ≥ 1.96), based on pointwise Z-score computation as described in the Methods. Relative to the normative reference, the patient exhibited reduced hip flexion during mid-swing, diminished knee flexion during swing-phase advancement, and decreased ankle dorsiflexion and plantarflexion during terminal stance and pre-swing, consistent with residual imbalance and impaired dynamic control despite near-normal muscle strength.

Despite normal strength, markerless gait analysis revealed distinct kinematic deviations. Hip flexion during early–mid swing (approximately 10–45% of the gait cycle) remained below the normative envelope, and peak knee flexion in late swing (60–80%) was reduced and delayed, indicating impaired limb advancement. Ankle kinematics showed diminished dorsiflexion in early stance and a blunted plantarflexion peak during terminal stance and pre-swing (55–70%), suggesting weak or delayed push-off despite normal MRC scores. Together, these patterns reflect altered vestibular–motor integration rather than isolated muscular weakness (Figure 5).

Overall, the patient exhibited subclinical deficits in swing-phase limb advancement, dynamic ankle control, and coordination that corresponded closely with her vestibular findings, providing biomechanical insight into persistent imbalance during subacute recovery.

### 3.3. Feasibility Outcomes

Both participants completed gait assessments without adverse events. Setup and calibration required less than five minutes, and each session—including data acquisition and rest breaks—was completed within a 20–30 min clinical visit. All videos were processed successfully using OpenCap’s cloud pipeline, yielding at least one analyzable gait cycle per participant without tracking failures.

Markerless kinematic data were obtained using only two smartphones, demonstrating the practicality of integrating this system into routine pediatric rehabilitation workflows. Processing time per trial was approximately 3–5 min. No participants required modifications to the protocol, and compliance was high.

## 4. Discussion

This two-case proof-of-concept study demonstrates that a smartphone-based markerless motion capture system (OpenCap) is a feasible, accessible, and clinically informative tool for quantifying gait impairment and recovery in pediatric Guillain–Barré syndrome (GBS). To our knowledge, this is the first study to demonstrate not only the feasibility but also the clinical interpretability and rehabilitation responsiveness of smartphone-based markerless gait analysis in pediatric GBS, providing detailed characterization of gait deviations and recovery-related kinematic changes that are not captured by conventional clinical measures. Traditional assessments of GBS rely heavily on subjective or low-resolution clinical measures—such as the MRC Sum Score, FAC, BBS, and timed functional tests—which may not detect subtle biomechanical abnormalities or emerging recovery patterns during rehabilitation [4,8]. In contrast, markerless kinematic data obtained through OpenCap provided objective, high-resolution insights into joint coordination, limb advancement, and gait symmetry that closely reflected underlying neuromuscular and sensory recovery processes.

To illustrate these potential advantages in real clinical scenarios, we analyzed two pediatric GBS cases with distinct recovery trajectories. Case 1 (treatment-related fluctuation) illustrated the ability of OpenCap to track longitudinal improvements in gait mechanics. Progressive increases in hip and knee flexion during swing and restoration of ankle rocker progression paralleled gains in strength and functional independence, supporting prior work demonstrating that knee flexion amplitude and ankle power generation are robust indicators of gait recovery in neuropathic conditions. The serial, time-normalized visualizations enabled by OpenCap offer clinicians a dynamic method of tracing kinematic trajectories—an advantage also highlighted in previous markerless gait studies in pediatric neuromuscular and orthopedic populations [23,24,25].

In contrast, Case 2 (AIDP with vestibular dysfunction) highlighted the potential for markerless motion capture to uncover biomechanical deficits not detectable through conventional strength-based measures [26]. Despite full MRC recovery, the patient demonstrated reduced hip and knee flexion during swing and impaired ankle dorsiflexion and plantarflexion during late stance—kinematic signatures consistent with instability and altered sensorimotor integration rather than weakness [27]. Similar discrepancies between clinical and biomechanical recovery have been reported in pediatric ataxia, peripheral nerve injury, and vestibular disorders, emphasizing the need for quantitative gait assessment tools that are sensitive to both motor and sensory contributors to gait impairment [6,27,28,29]. In this context, the observed reduction in hip and knee flexion during swing and the blunted ankle rocker pattern in Case 2 may reflect adaptive motor strategies associated with vestibular dysfunction. Disruption of vestibulo-spinal and vestibulo-motor pathways has been shown to promote cautious gait patterns characterized by reduced limb excursion, diminished push-off, and increased reliance on conservative stability strategies to minimize perceived postural threat during dynamic walking [30,31,32]. Such adaptations are consistent with altered dynamic balance control rather than primary muscle weakness in this patient [33].

The feasibility of smartphone-based markerless motion capture demonstrated in this study aligns with the growing recognition that traditional marker-based systems, while accurate, are limited by cost, lengthy preparation time, and poor adaptability to routine pediatric clinical environments [13,14,23,25]. An additional advantage of the proposed approach is its non-intrusive setup, which requires no physical markers, wearable sensors, or specialized clothing, thereby minimizing participant burden and facilitating more natural movement, an especially important consideration in pediatric rehabilitation settings. Markerless systems such as OpenCap, OpenPose, and MediaPipe-based pipelines have shown strong validity for lower-limb kinematics in walking, running, and clinical assessments [18,24,34,35]. The successful acquisition of high-quality kinematic data in children—including one in the subacute phase and another undergoing active recovery—supports previous findings that markerless motion capture is practical in real-world outpatient and inpatient settings. The noninvasive nature of these systems is particularly advantageous for pediatric rehabilitation where tolerance, engagement, and efficiency are crucial.

Beyond feasibility, markerless systems offer novel opportunities for precision rehabilitation, data sharing, and musculoskeletal simulation. OpenCap’s open-source workflow and compatibility with OpenSim provide a reproducible pipeline for clinicians and researchers to model joint kinetics, muscle forces, and neuromuscular impairment profiles [17,19]. Such integration aligns with recent initiatives in digital health and pediatric rehabilitation to incorporate quantitative biomarkers, cloud-based data repositories, and AI-driven analytics into routine care [18,36]. Future applications may include automated gait quality scoring, individualized recovery prediction, and remote rehabilitation monitoring, advancing the broader vision of technology-enabled pediatric neurorehabilitation [15,36]. Building on these broader opportunities, the present study focused on brief straight-walking trials conducted in a clinical corridor to establish the feasibility and initial clinical utility of smartphone-based markerless gait analysis. Although this controlled approach enabled repeatable and interpretable kinematic assessment suitable for a proof-of-concept investigation, it did not capture room-level location or continuous indoor mobility. Integration of markerless gait analysis with indoor localization techniques therefore represents an important next step, as it may enable context-aware assessment of functional mobility across everyday environments such as homes, schools, and therapy spaces [37,38]. Future work will explore the feasibility of combining vision-based gait kinematics with indoor localization to support continuous, ecologically valid monitoring in pediatric neurorehabilitation.

The present study focused on brief straight-walking trials conducted in a clinical corridor to establish the feasibility and clinical utility of smartphone-based markerless gait analysis. Although this approach enabled controlled, repeatable kinematic assessment, it did not capture room-level location or continuous indoor mobility. Integration of markerless gait analysis with indoor localization techniques represents an important future direction, as it may enable context-aware assessment of functional mobility across everyday environments such as homes, schools, and therapy spaces. Future work will explore the feasibility of combining vision-based gait kinematics with indoor localization to support continuous, ecologically valid monitoring in pediatric neurorehabilitation.

Several limitations should be acknowledged. First, this was a small, exploratory study involving only two cases, and the results should be interpreted qualitatively rather than statistically. Although normative reference data were collected under similar self-selected walking conditions, exact matching of walking speed between patients and controls was not enforced, and differences in gait speed may have influenced the magnitude of observed joint kinematic deviations. Second, while OpenCap has been validated against marker-based motion capture systems, minor errors in three-dimensional joint estimation might persist, particularly in out-of-plane movements or in children with atypical postures. Third, this study focused primarily on kinematic analysis and did not include kinetic measurements such as ground reaction forces or joint moments, nor electromyography, which limits the ability to directly quantify propulsion, loading patterns, and underlying neuromuscular mechanisms during gait. While beyond the scope of this feasibility investigation, future studies may integrate smartphone-based markerless kinematics with portable force plates, wearable inertial sensors, or complementary modalities such as plantar pressure and vestibular measures to enable more comprehensive multimodal gait assessment in pediatric Guillain–Barré syndrome.

## 5. Conclusions

This study provides preliminary evidence that smartphone-based markerless motion capture can sensitively identify both recovery-related improvements and residual gait deficits in pediatric GBS. The integration of an accessible, low-burden technology with quantitative biomechanical analysis may enhance clinical decision-making, support individualized rehabilitation planning, and improve functional outcome assessment in children with neurological impairments.

## Data Availability

The data supporting the findings of this study, including raw OpenCap motion files, processed kinematic outputs, and all analysis code, are available in a publicly accessible repository. The Zenodo repository contains raw video-derived pose and keypoint data, time-normalized joint angle files, and Python scripts used for gait cycle extraction, Z-score computation, and figure generation. These materials can be found on Zenodo at https://doi.org/10.5281/zenodo.17501242.

## Abbreviations

GBS	Guillain–Barré syndrome
AIDP	Acute inflammatory demyelinating polyneuropathy
TRF	Treatment-related fluctuation
IVIG	Intravenous immunoglobulin
MRC	Medical Research Council
FAC	Functional Ambulation Category
BBS	Berg Balance Scale
K-MBI	Korean Modified Barthel Index
10MWT	10-Meter Walk Test
TUG	Timed Up and Go
VOG	Video-oculography
VEMP	Vestibular-evoked myogenic potential
SD	Standard deviation
IK	Inverse kinematics
ROM	Range of motion

## References

[1] van Doorn P.A., Van den Bergh P.Y.K., Hadden R.D.M., Avau B., Vankrunkelsven P., Attarian S., Blomkwist-Markens P.H., Cornblath D.R., Goedee H.S., Harbo T. (2023). European Academy of Neurology/Peripheral Nerve Society Guideline on Diagnosis and Treatment of Guillain-Barré Syndrome. Eur. J. Neurol..

[2] Leonhard S.E., Mandarakas M.R., Gondim F.A.A., Bateman K., Ferreira M.L.B., Cornblath D.R., van Doorn P.A., Dourado M.E., Hughes R.A.C., Islam B. (2019). Diagnosis and Management of Guillain-Barré Syndrome in Ten Steps. Nat. Rev. Neurol..

[3] Tekgul H., Serdaroglu G., Tutuncuoglu S. (2003). Outcome of Axonal and Demyelinating Forms of Guillain-Barré Syndrome in Children. Pediatr. Neurol..

[4] Jin M., Zhao L., Liu J., Geng W., Zhao Z., Li C., Xue J., Sun S. (2021). Association Between the Rate of Treatment Response and Short-Term Outcomes in Childhood Guillain-Barré Syndrome. Front. Neurol..

[5] Kiper P., Chevrot M., Godart J., Cieślik B., Kiper A., Regazzetti M., Meroni R. (2025). Physical Exercise in Guillain-Barré Syndrome: A Scoping Review. J. Clin. Med..

[6] Hulleck A.A., Mohan D.M., Abdallah N., El Rich M., Khalaf K. (2022). Present and Future of Gait Assessment in Clinical Practice: Towards the Application of Novel Trends and Technologies. Front. Med. Technol..

[7] Bhagwat A.P., Sharath H.V., Warghat P.A. (2024). Effect of Paediatric Rehabilitation in Children with Guillain-Barré Syndrome: A Case Series. Cureus.

[8] Estublier B., Colineaux H., Arnaud C., Cintas P., Baudou E., Chaix Y., Rivier F., Biotteau M., Meyer P., Cheuret E. (2024). Long-Term Outcomes of Paediatric Guillain-Barré Syndrome. Dev. Med. Child Neurol..

[9] Turan Z., Topaloglu M., Taskiran O.O. (2020). Medical Research Council-Sumscore: A Tool for Evaluating Muscle Weakness in Patients with Post-Intensive Care Syndrome. Crit. Care.

[10] Baker R., Esquenazi A., Benedetti M.G., Desloovere K. (2016). Gait Analysis: Clinical Facts. Eur. J. Phys. Rehabil. Med..

[11] Böhm H., Stebbins J., Kothari A., Dussa C.U. (2024). Dynamic Gait Analysis in Paediatric Flatfeet: Unveiling Biomechanical Insights for Diagnosis and Treatment. Children.

[12] Faccioli S., Cavalagli A., Falocci N., Mangano G., Sanfilippo I., Sassi S. (2023). Gait Analysis Patterns and Rehabilitative Interventions to Improve Gait in Persons with Hereditary Spastic Paraplegia: A Systematic Review and Meta-Analysis. Front. Neurol..

[13] Yang J., Park K. (2024). Improving Gait Analysis Techniques with Markerless Pose Estimation Based on Smartphone Location. Bioengineering.

[14] Kanko R.M., Laende E.K., Strutzenberger G., Brown M., Selbie W.S., DePaul V., Scott S.H., Deluzio K.J. (2021). Assessment of Spatiotemporal Gait Parameters Using a Deep Learning Algorithm-Based Markerless Motion Capture System. J. Biomech..

[15] Lam W.W.T., Tang Y.M., Fong K.N.K. (2023). A Systematic Review of the Applications of Markerless Motion Capture (MMC) Technology for Clinical Measurement in Rehabilitation. J. Neuroeng. Rehabil..

[16] Mathis A., Mamidanna P., Cury K.M., Abe T., Murthy V.N., Mathis M.W., Bethge M. (2018). DeepLabCut: Markerless Pose Estimation of User-Defined Body Parts with Deep Learning. Nat. Neurosci..

[17] Uhlrich S.D., Falisse A., Kidziński Ł., Muccini J., Ko M., Chaudhari A.S., Hicks J.L., Delp S.L. (2023). OpenCap: Human Movement Dynamics from Smartphone Videos. PLoS Comput. Biol..

[18] Cerfoglio S., Storniolo J.L., de Borba E.F., Cavallari P., Galli M., Capodaglio P., Cimolin V. (2025). Smartphone-Based Gait Analysis with OpenCap: A Narrative Review. Biomechanics.

[19] Seth A., Hicks J.L., Uchida T.K., Habib A., Dembia C.L., Dunne J.J., Ong C.F., DeMers M.S., Rajagopal A., Millard M. (2018). OpenSim: Simulating Musculoskeletal Dynamics and Neuromuscular Control to Study Human and Animal Movement. PLoS Comput. Biol..

[20] Rajagopal A., Dembia C.L., DeMers M.S., Delp D.D., Hicks J.L., Delp S.L. (2016). Full-Body Musculoskeletal Model for Muscle-Driven Simulation of Human Gait. IEEE Trans. Biomed. Eng..

[21] Lai A.K.M., Arnold A.S., Wakeling J.M. (2017). Why are Antagonist Muscles Co-activated in My Simulation? A Musculoskeletal Model for Analysing Human Locomotor Tasks. Ann. Biomed. Eng..

[22] Uhlrich S.D., Jackson R.W., Seth A., Kolesar J.A., Delp S.L. (2022). Muscle Coordination Retraining Inspired by Musculoskeletal Simulations Reduces Knee Contact Force. Sci. Rep..

[23] Scataglini S., Abts E., Van Bocxlaer C., Van den Bussche M., Meletani S., Truijen S. (2024). Accuracy, Validity, and Reliability of Markerless Camera-Based 3D Motion Capture Systems versus Marker-Based 3D Motion Capture Systems in Gait Analysis: A Systematic Review and Meta-Analysis. Sensors.

[24] Turner J.A., Chaaban C.R., Padua D.A. (2024). Validation of OpenCap: A Low-Cost Markerless Motion Capture System for Lower-Extremity Kinematics during Return-to-Sport Tasks. J. Biomech..

[25] Horsak B., Eichmann A., Lauer K., Prock K., Krondorfer P., Siragy T., Dumphart B. (2023). Concurrent Validity of Smartphone-Based Markerless Motion Capturing to Quantify Lower-Limb Joint Kinematics in Healthy and Pathological Gait. J. Biomech..

[26] Kanko R.M., Laende E.K., Davis E.M., Selbie W.S., Deluzio K.J. (2021). Concurrent Assessment of Gait Kinematics Using Marker-Based and Markerless Motion Capture. J. Biomech..

[27] Horlings C.G.C., Carpenter M.G., Honegger F., Allum J.H.J. (2009). Vestibular and Proprioceptive Contributions to Human Balance Corrections: Aiding These with Prosthetic Feedback. Ann. N. Y. Acad. Sci..

[28] Smani A., Lee S., Spinner M., Barbuto S. (2025). Changes in Gait After Training for Individuals with Cerebellar Ataxia. Arch. Rehabil. Res. Clin. Transl..

[29] Smith M., Kurian M.A. (2018). Neurological Gait Disorders in Childhood. Paediatr. Child Health.

[30] Iles J.F., Baderin R., Tanner R., Simon A. (2007). Human Standing and Walking: Comparison of the Effects of Stimulation of the Vestibular System. Exp. Brain Res..

[31] Schniepp R., Möhwald K., Wuehr M. (2017). Gait Ataxia in Humans: Vestibular and Cerebellar Control of Dynamic Stability. J. Neurol..

[32] Brandt T., Dieterich M., Strupp M. (2013). Vertigo and Dizziness: Common Complaints.

[33] Horak F.B. (2006). Postural Orientation and Equilibrium: What Do We Need to Know about Neural Control of Balance to Prevent Falls?. Age Ageing.

[34] Lima Y.L., Collings T., Hall M., Bourne M.N., Diamond L.E. (2024). Validity and Reliability of Trunk and Lower-Limb Kinematics during Squatting, Hopping, Jumping and Side-Stepping Using OpenCap Markerless Motion Capture Application. J. Sports Sci..

[35] Min Y.-S., Jung T.-D., Lee Y.-S., Kwon Y., Kim H.J., Kim H.C., Lee J.C., Park E. (2024). Biomechanical Gait Analysis Using a Smartphone-Based Motion Capture System (OpenCap) in Patients with Neurological Disorders. Bioengineering.

[36] Yang H.S. (2024). Advances in Markerless Motion Capture Systems: A Review of OpenCap and Its Applications. Asian J. Kinesiol..

[37] Thakur N., Han C.Y. (2021). Multimodal Approaches for Indoor Localization for Ambient Assisted Living in Smart Homes. Information.

[38] Tsanousa A., Xefteris V.-R., Meditskos G., Vrochidis S., Kompatsiaris I. (2021). Combining RSSI and Accelerometer Features for Room-Level Localization. Sensors.

